# The role of religiosity and religious participation in the relationship between depressive symptoms and cognitive impairment among older Indian adults

**DOI:** 10.1038/s41598-022-14744-3

**Published:** 2022-07-13

**Authors:** T. Muhammad

**Affiliations:** Department of Family & Generations, International Institute for Population Sciences, Mumbai, India 400088

**Keywords:** Geriatrics, Health policy, Public health

## Abstract

Due to different nature of social engagements of older adults in South Asian countries specially attributed to the traditional family-based care and support, beneficial effects of religiosity and religious involvement on mental health and cognitive function in older age might be different than those in the Western world. Yet, there is a paucity of research in these countries on the role of religion in moderating the relationship between late life depression and cognition. This study explored the association of depressive symptoms with cognitive impairment and the moderating effects of religiosity and religious participation in those associations among older Indian adults. A cross-sectional study was conducted on data that were drawn from the Longitudinal Ageing Study in India wave-1, collected during 2017–2018. The sample size comprised of 31,464 older adults aged 60 years and above. Shortened 10-item Centre for Epidemiologic Studies Depression Scale was used to measure depressive symptoms. Items from the Mini-Mental State Examination and the cognitive module of the China Health and Retirement Longitudinal Study and the Mexican Health and Aging Study were adapted for measuring cognitive impairment. Moderated multiple linear regression models were used to test the research hypotheses of the study. The proportion of older adults who reported religion as less important to them was 21.24%, whereas, only 19.31% of the respondents participated in religious activities. The mean score of cognitive impairment (on a scale of 0–43) in the current sample was 19.43 [confidence interval (CI): 19.32–19.53] among men and 23.55 [CI: 23.44–23.66] among women. Older adults with depressive symptoms had significantly higher likelihood of cognitive impairment [aCoef: 0.18, CI: 0.16–0.20] in comparison to older adults with no depressive symptoms. Older individuals who were religious were significantly less likely to have cognitive impairment [aCoef: − 0.43, CI: − 0.61 to − 0.25] than their non-religious counterparts. Compared to older adults who did not participate in religious activities, those who participated in religious activities were less likely [aCoef: − 0.52, CI: − 0.69 to − 0.34] to have cognitive impairment. Further, significant moderating effects of religiosity and religious participation in the relationship between depressive symptoms and cognitive impairment were observed. The current study contributes to advancing knowledge about the mental health benefits of religiosity and religious participation by focusing on older adults in India who culturally have limited chances to participate in social activities. The findings suggest that older adults with depressive symptoms may participate in religious activities which may reduce their chances of cognitive impairment. This protective effect of religiosity and religious participation on late life cognitive health has important implications for promoting alternative social support mechanisms for older adults in terms of enhancing their mental wellbeing and contributing to active aging.

## Introduction

Depression is a serious mood disorder that can affect the way individuals feel, act and think^1^. Unlike the situation in developed countries where older people often show lower depressive symptoms than younger persons, prevalence of depression in older Indian adults is shown to be much greater than younger population^2,3^. A meta-analysis of 51 Indian studies revealed that the prevalence of depression among older adults ranged from 29.3 to 39.7%, which is comparable to other low and middle-income countries^4^. Depressive symptoms in older age are particularly challenging since they can affect older individuals’ cognitive process and influence their social interaction^5^.

Previous research has demonstrated the linkage between late-life depression and cognitive impairment and suggested that depression may be a prodrome to dementia among older adults^6,7^. Similarly, participation in social activities is associated with mental health outcomes and direction of those associations varies by types of activity performed. According to the previous research, religious activity protects against mental illnesses, while political activity increases the risk for mental disorders^8^. Earlier evidence also suggests that depressed people have least motivation to engage in social activities and often try to avoid social contacts^9^, which in turn may lead to their cognitive deficits. Social isolation is more frequent among those people due to difficulty in maintaining friendships and narrowing their social network that may prompt even more negative mental health consequences^10–12^.

Studies have documented the positive association of religious involvement with various health outcomes^13,14^, and reduced mortality and increased longevity^15–17^. The importance of faith-based treatment of depression and mental health interventions such as faith-adapted psychological and cognitive therapies has been highlighted in multiple studies^18–22^. Several cross-sectional studies in clinical settings found that religiosity is positively related to better mental health and can minimize the problems of depression and cognitive impairment which are common complications in older patients^23,24^. A review concluded that positive religious coping is associated with fewer symptoms of depression both in longitudinal and cross-sectional studies in clinical and community settings^25^. Further, a community-based study in Korea revealed that older subjects who frequently participated in religious activity were less likely to be depressed^26^. Study reported that when older believers develop mental problems such as cognitive and emotional frustrations, they resort to church sacraments and prayers which act as relievers^27^. On the other hand, Social elements of religious activities may promote mental stimulation and build cognitive reserve capacity and at the same time, social disengagement may be a risk factor for cognitive decline among older individuals^28,29^.

In Rowe and Kahn’s successful aging model^30^, religious involvement is included as a factor which may facilitate improved physical and cognitive health outcomes among older adults. In terms of strengthening the successful aging model, Crowther et al.^31^ further suggested to add the factor of positive spirituality as it encourages behaviors such as meditation and prayer that can also reduce the physiological stress of older people. Several types of non-material support such as self-control and sense of belonging within the religious groups may be particularly important for older individuals who are socioeconomically disadvantaged^32^. A recent study has found that religious involvements allowed older adults to cope with stress, enhance the support networks and keep their minds active^33^.

**Figure 1:**
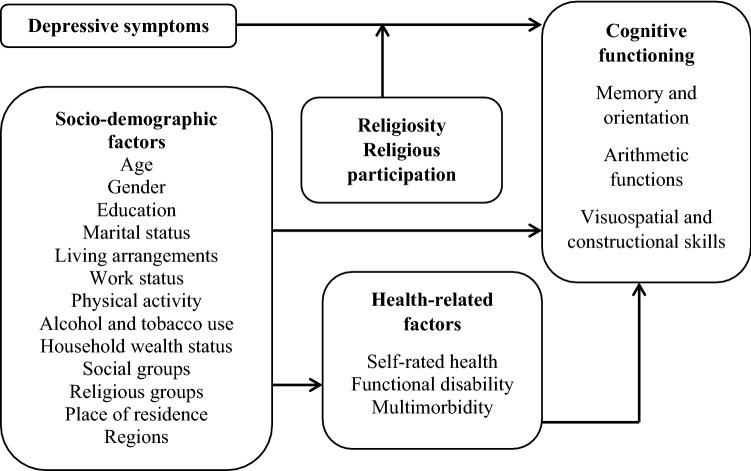
Conceptual model for the study.

Importantly, religion and religiosity have multiple dimensions such as beliefs, rituals and devotional activities, and social relationships^34,35^. However, knowledge regarding the linkage between religiosity, participation in religious activities and cognitive functions in older people is limited. Due to different nature of social engagements of older adults in South Asian countries specially attributed to the traditional family-based care and support, and lack of formal welfare system, beneficial effects of religiosity and religious involvement on mental health and cognitive function in older age might be different than those in the Western world. Yet, there is a paucity of research in these countries that empirically assesses the role of religion in moderating the relationship between late life depression and cognition. Therefore, in this study, I explore the associations of depressive symptoms with cognitive impairment and the moderating effects of religiosity and religious participation in those associations after controlling for several socioeconomic and health-related factors. Based on the abovementioned literature, a conceptual model has been developed and summarized in Fig. 1.

## Methods

### Study design and sample

**Figure 2:**
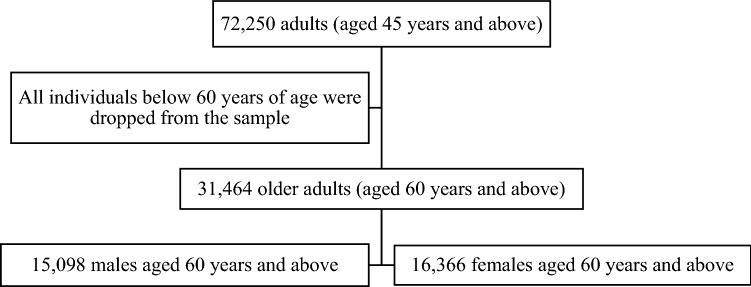
Sample selection criteria for this study.

A cross-sectional study design was adopted and the data were drawn from the Longitudinal Ageing Study in India (LASI) wave-1, collected during 2017–2018. The LASI is a nationally representative survey of 72,250 individuals aged 45 and above from all states and union territories of India. The survey was designed to collect data on the health status and socioeconomic well-being of older adults in the country. The current study is conducted on eligible respondents defined as older adults aged 60 years and above. Thus, the total sample size comprised of 31,464 older adults (men-15,098 and women-16,366). The sample selection procedure of this study is summarized in Fig. 2.

### Procedure

The survey adopted a three-stage sampling design in rural areas and a four-stage sampling design in urban areas. In each state and union territory, the first stage involved the selection of Primary Sampling Units (PSUs), that is, sub-districts (Tehsils/Talukas), and the second stage involved the selection of villages in rural areas and wards in urban areas in the selected PSUs. In rural areas, households were selected from selected villages in the third stage. However, sampling in urban areas involved an additional stage. Specifically, in the third stage, one Census Enumeration Block (CEB) was randomly selected in each urban area. In the fourth stage, households were selected from this CEB. The goal was to select a representative sample in each stage of sample selection. Further, an individual survey schedule was administered to each consenting respondent aged 45 and over and their spouses (irrespective of age) in the sampled households. In addition, the LASI included an individual module on biomarkers and direct health examination. The detailed methodology, with the complete information on the survey design and data collection, was published in the survey report^36^.

### Measures

#### Outcome variable

##### Cognitive impairment

The LASI collected the information related to different cognition measures in several dimensions including memory, orientation, arithmetic functioning, and visuospatial and constructional ability. The survey adapted these measures from the Mini-Mental State Examination (MMSE)^37^, and the cognitive module of the China Health and Retirement Longitudinal Study (CHARLS), and the Mexican Health and Aging Study (MHAS)^38,39^. Thus, the cognitive functions in the current study are based on the scoring of the following cognitive sub-domains: immediate word recall (0–10 points), and delayed word recall (0–10 points); orientation related to time (0–4 points), and place (0–4 points); arithmetic ability based on serial 7 s’ subtraction task (0–5 points), a task involving two computations (0–2) and backward counting from 20 (0–2 points) ^38^; visuospatial and constructional skills based on paper folding (folding a piece of paper according to instructions) (0–3) and pentagon drawing (drawing intersecting circles) (0–1); and object naming (0–2)^39^. The respondents who received assistance during the cognition module (proxy informants) were excluded from the analysis. The composite score ranged between 0 and 43, and a higher score indicates better cognitive functioning. The scoring was reversed to assess cognitive impairment among older adults in this study.

#### Main explanatory variables

##### Depressive symptoms

A shortened 10-item Centre for Epidemiologic Studies Depression Scale (CES-D) with four response items was used in the LASI to assess depressive symptoms. The 10 items included seven negative symptoms (trouble concentrating, feeling depressed, low energy, fear of something, feeling alone, bothered by things, and everything is an effort), and three positive symptoms (feeling happy, hopeful, and satisfied) which is a modified version of the CES-D scale developed by Andresen^40^. Response options included “rarely or never” (< 1 day), “sometimes” (1 or 2 days), “often” (3 or 4 days), and “most or all of the time” (5–7 days) in a week prior to the interview. Scoring was reversed for positive symptoms. The overall CES-D score ranged from zero to 30 and a higher score indicated higher depressive symptoms. The coefficient alpha for the CES-D in this study was 0.71, which was comparable to the values obtained in previous reliability studies^41,42^.

##### Religious participation

Religious participation was assessed using the question ‘how often do you engage in the following religious activities? (a) done *pooja* or prayer?, (b) attended religious services (at temple/mosque/church, etc.)?, and (c) involved yourself in *satsang/bhajan/kirtan*/or any religious gathering?. The responses were “every day” , “more than once a week”, “once a week”, “one to three times a month”, “one or more times a year” and “not at all”. Further, it was recoded as 0 ‘no’ (less than once a month) and 1 ‘yes’ (at least once a month). Thus, religious participation in this study refers to participating in all of the above activities at least once a month.

##### Religiosity

Religious participation is discussed as an indicator of social solidarity rather than an aspect of religiosity^43^. Thus, the religiosity of older adults was also considered in the study as a moderator in the association between depressive symptoms and cognitive impairment. It was assessed using the question ‘How important would you say religion is in your life? And the responses were “very important”, “somewhat important” and “not too important”. This single item subjective report was validated in measuring religious/spiritual importance ranking highest among a diverse latent factor structure^44^. It was recoded as 0 ‘no’ (not too/somewhat important) and 1 ‘yes’ (very important)^45,46^.

### Individual factors

The following socio-demographic variables that were related to both late life mental health^47^ and cognition^48^ were included in the analysis. Age was categorized into groups of 60–69 years, 70–79 years, and 80+ years. Sex was coded as male and female. Marital status was coded as currently married and unmarried. Currently unmarried included widowed/divorced/separated/never married. Types of living arrangements were grouped into ‘living alone’, ‘living with spouse’ and ‘other living arrangements’. Educational status was coded as no education, primary, secondary and higher. Working status was coded as never worked, currently not working, currently working, and retired.

### Behavioral and health-related factors

Prior research has identified several lifestyle behaviours that are likely to be associated with late life depression and cognitive functions such as physical activity^26,48^, tobacco use and alcohol consumption^49,50^. Physical activity was coded as inactive, only moderate, only vigorous and both moderate and vigorous activities^51^. Current tobacco use (smoked/smokeless) and heavy episodic drinking were coded as no and yes.

Health covariates were selected based on their clinical importance reported in previous studies^52–55^, and significance level in the bivariate analysis. Self-rated health (SRH) was coded as good which includes very good, good and fair whereas, poor includes poor and very poor. Multi-morbidity condition refers to the presence of two or more chronic diseases which include hypertension, chronic heart diseases, stroke, any chronic lung disease, diabetes, cancer or malignant tumor, any bone/joint disease and neurological/psychiatric diseases. The diseases were self-reported^56^ and assessed through the question “Has any health professional ever diagnosed you with the following chronic conditions or diseases?” Both activities of daily living (ADL) and Instrumental ADL (IADL) functioning were coded as high and low; high representing the absence of any functional disability and low representing at least one disability. ADL is a term used to refer to normal daily self-care activities (such as movement in bed, changing position from sitting to standing, feeding, bathing, dressing, grooming, personal hygiene etc.) IADL functions are those which are not necessarily related to fundamental functioning of a person, but they let an individual live independently in a community^57,58^.

### Household/community-related factors

Following socioeconomic and contextual variables were also included in this study^55,59^. The monthly per-capita consumption expenditure (MPCE) quintile was assessed using household consumption data. Sets of 11 and 29 questions on the expenditures on food and non-food items, respectively, were used to canvas the sample households. Food expenditure was collected based on a reference period of 7 days, and non-food expenditure was collected based on reference periods of 30 days and 365 days. Food and non-food expenditures have been standardized to the 30 days reference period. The MPCE is computed and used as the summary measure of consumption^36^. The variable was available in the survey data as divided into five quintiles of poorest, poor, middle, rich and richest. Religion was recoded as Hindu, Muslim, Christian and Others (Sikh, Buddhist, Jain, etc.). Caste was recoded as Scheduled Castes/Scheduled Tribes (SC/ST), Other Backward Classes (OBC), and others (mostly the higher caste groups). The residential status was coded as urban and rural. The regions were coded as North, Central, East, Northeast, West, and South.

### Statistical approach

In this study, descriptive statistics were reported to present the sample distribution and the mean scores of cognitive impairment along with 95% confidence interval (CI) across the explanatory variables. A two-way scatter plot was presented on the relationship between two continuous variables of depressive symptoms and cognitive impairment. Further, moderated linear regression models were used to fulfil the objective of the study. The results were summarized in the form of unadjusted and adjusted coefficients (uCoef and aCoef) with a 95% CI and standardized beta coefficients. Individual weights were used to make the estimates nationally representative. For all the analyses, Stata version 15 has been used^60^. Variance inflation factor (VIF) was generated in Stata to check the multicollinearity and there was no violation of the assumption of no multicollinearity in the variables used^61,62^.

Along with unadjusted and adjusted (for all the individual-, health- and household/community-related variables) models, the multivariable analysis provides two models to explain the moderated regression estimates with interaction terms^63^ that examine the moderating role of religiosity, religious participation and depressive symptoms on cognitive impairment among older adults. Additionally, a coefficient plot of multiple interactions between religion, religiosity, religious participation and depressive symptoms on cognitive impairment is provided.

### Ethical approval and consent to participate

The survey agencies involved in the field survey for the data collection of LASI have collected prior informed consent (written and verbal) from all the participants. The Indian Council of Medical Research (ICMR) extended the necessary guidance and ethical approval for conducting the LASI survey.

All methods were carried out in accordance with relevant guidelines and regulations by the ICMR.

## Results

### Socio-economic and health profile of older adults

Table 1 presents the socio-economic and health profile of older adults. The proportion of older adults who reported religion as less important to them was 21.24%, whereas, only 17.19% of the respondents participated in all the religious activities at least once in a month. The share of the sample in the age group of 80 years and above was 11.29%. In the study sample, 52.55% of older adults were females. A proportion of 74.02% were illiterate or had a primary level education, whereas, only 7.74% were highly educated. Besides, 29.45% of the study participants belonged to urban areas against 70.55% who were rural residents.

**Table 1:** Socio-economic and health profile of older adults in India.

**Background characteristics**	**Missing cases**	**w%**	**Frequency**
**Age (in years)**	–		
60–69		58.51	18,974
70–79		30.20	9101
80+		11.29	3389
**Sex**	–		
Male		47.45	15,098
Female		52.55	16,366
**Marital status**	–		
Currently in marital union		61.63	19,920
Not in marital union		38.37	11,544
**Living arrangement **	–		
Alone		5.68	1622
With spouse		20.33	6215
Others		73.99	23,627
**Educational status**	–		
No		56.52	16,889
Primary		17.50	5840
Secondary		18.24	6106
Higher		7.74	2629
**Work status**	–		
Never worked		26.43	8784
Currently not working		36.45	10,990
Currently working		29.87	8997
Retired		7.25	2693
**Religiosity**	559		
No		21.24	6081
Yes		78.76	24,824
**Religious participation**	54		
No		82.81	25,879
Yes		17.19	5531
**Physical activity**	455		
No		74.99	23,430
Moderate		6.92	2083
Vigorous		15.49	4767
Both		2.59	729
**Current tobacco use (smoked/smokeless)**	252		
No		66.30	21,369
Yes		33.70	9843
**Heavy episodic drinking**	257		
No		94.65	29,163
Yes		5.35	2044
**SRH**	666		
Good		75.79	23,685
Poor		24.21	7113
**Multimorbid**	91		
No		75.95	23,576
Yes		24.05	7797
**ADL functioning**	128		
High		76.23	24,642
Low		23.77	6694
**IADL functioning**	169		
High		51.64	17,449
Low		48.36	13,846
**MPCE quintile**	–		
Poorest		21.70	6484
Poorer		21.71	6477
Middle		20.95	6416
Richer		19.19	6170
Richest		16.45	5917
**Religion**	–		
Hindu		82.22	23,037
Muslim		11.28	3731
Christian		2.86	3150
Others		3.64	1546
**Caste**	–		
SC/ST		27.10	10,313
OBC		45.20	11,886
Others		27.70	9,265
**Residential status**	–		
Urban		29.45	10,739
Rural		70.55	20,725
**Region**	–		
North		12.59	5812
Central		20.95	4262
East		23.64	5757
Northeast		2.97	3752
South		22.68	7578
West		17.17	4303
**Total**		100	31,464

w%: weighted percentage prevalence to provide national population estimates; Counts are unweighted; SRH: Self-Rated Health; ADL: Activities of daily living; IADL: Instrumental activities of daily living; MPCE: Monthly per capita consumption expenditure; SC/ST: Scheduled caste/Scheduled tribe; OBC: Other backward classes.

### Mean score of cognitive impairment among older Indian adults by background characteristics

**Figure 3:**
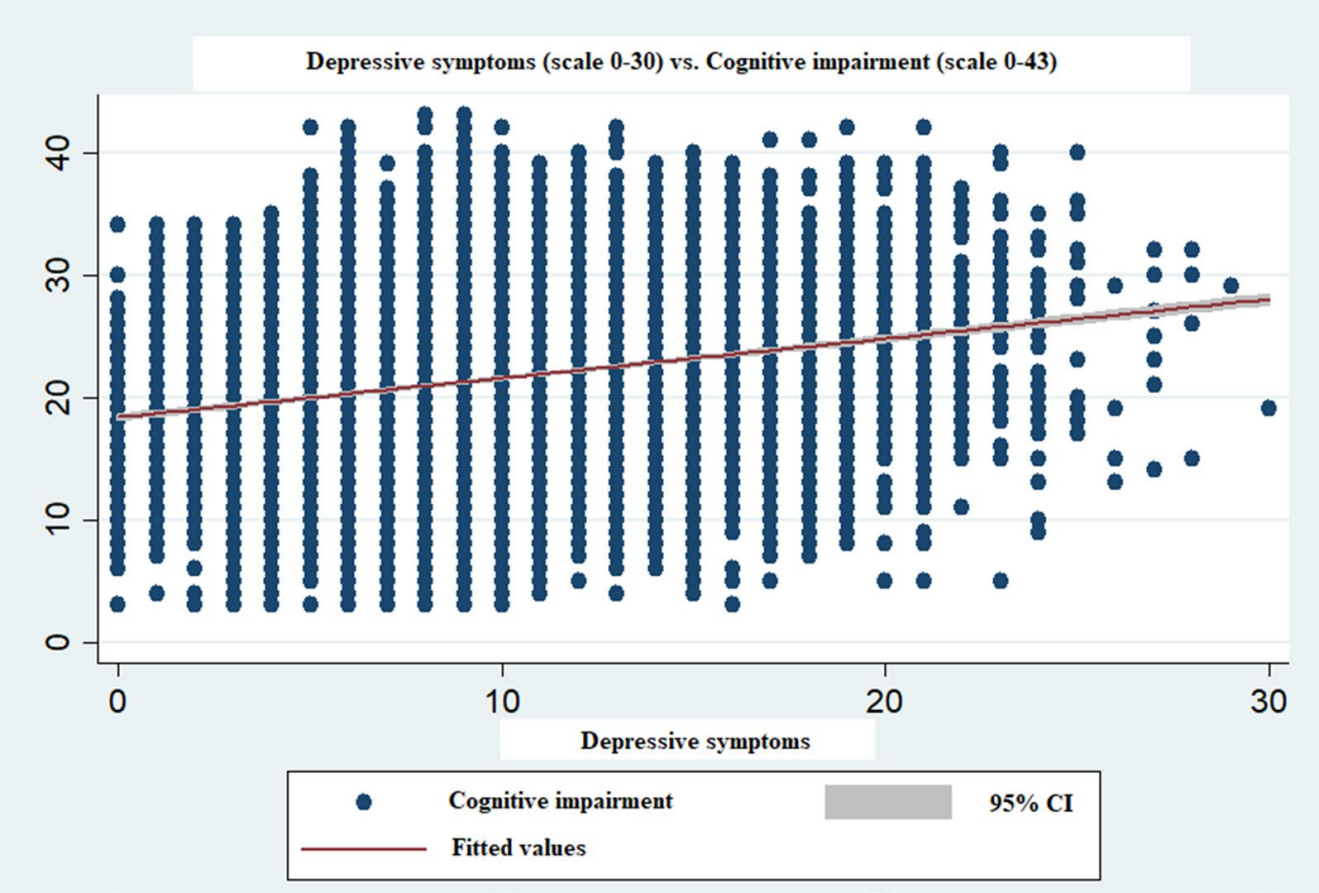
Scatter plot of the significant association (*p* < 0.001) between depressive symptoms and cognitive impairment among older adults. *Notes* Score of CES-D scale ranges from 0 to 30; Composite cognitive impairment scale ranges from 0 to 43.

Table 2 depicts the mean score of cognitive impairment (total score ranges 0–43) among older men and women across the explanatory variables. The overall mean score of cognitive impairment for the current sample was 19.43 [CI 19.32–19.53] among men and 23.55 [CI 23.44–23.66] among women. It was found that mean score of cognitive impairment among older men (19.17 vs 20.55) and women (23.25 vs 24.81) who were religious were lower than those who were not religious. On the other hand, mean score of cognitive impairment in older men (18.39 vs 19.68) and women (22.14 vs 23.84) who participated in religious activities were lower in comparison to that of those who did not participate in religious activities. Further, the mean score of cognitive impairment was highest among oldest-old individuals (22.85 in men and 27.44 in women). Older men (23.75) and women (26.03) who were illiterate had a substantially higher mean score of cognitive impairment than their educated counterparts. Figure 3 shows the scatter plot for the significant association (*p* < 0.001) between depressive symptoms and cognitive impairment among older adults.

**Table 2:** Mean score of cognitive impairment (score ranges from 0 to 43) by background characteristics among older adults.

Variables	Men	95% CI	Women	95% CI
**Religiosity**				
No	20.55	20.28–20.81	24.81	24.54–25.07
Yes	19.17	19.05–19.29	23.25	23.13–23.37
**Religious participation**				
No	19.68	19.56–19.8	23.84	23.72–23.96
Yes	18.39	18.17–18.62	22.14	21.86–22.41
**Age (in years)**				
60–69	18.64	18.51–18.77	22.51	22.37–22.65
70–79	20.02	19.83–20.22	24.90	24.69–25.11
80 +	22.85	22.47–23.24	27.44	27.06–27.82
**Marital status**				
Currently in marital union	19.10	18.98–19.21	22.53	22.38–22.69
Not in marital union	21.03	20.76–21.3	24.53	24.37–24.69
**Living arrangement**				
Alone	20.22	19.48–20.95	24.48	24.08–24.89
With spouse	19.41	19.19–19.63	22.67	21.86–22.41
With others	19.4	19.28–19.53	23.64	23.51–23.77
**Educational status**				
No	23.75	23.58–23.91	26.03	25.92–26.14
Primary	19.84	19.65–20.03	21.24	21–21.48
Secondary	16.72	16.57–16.87	17.29	17.06–17.53
Higher	14.28	14.09–14.48	13.70	13.34–14.05
**Work status**				
Never worked	20.68	20.13–21.22	23.09	22.93–23.25
Currently not working	20.54	20.36–20.72	24.78	24.58–24.97
Currently working	19.54	19.39–19.7	23.86	23.62–24.09
Retired	16.14	15.92–16.37	16.19	15.46–16.92
**Physical activity**				
No	19.95	19.82–20.09	23.91	23.79–24.03
Moderate	17.12	16.81–17.44	20.74	20.26–21.22
Vigorous	19.40	19.17–19.63	23.17	22.85–23.48
Both	15.77	15.28–16.26	18.31	17.4–19.23
**Current tobacco use (smoked/smokeless)**				
No	18.61	18.46–18.76	23.13	23.01–23.26
Yes	20.30	20.08–20.51	24.96	24.7–25.23
**Heavy episodic drinking**				
No	19.23	19.12–19.35	23.49	23.38–23.6
Yes	20.86	20.55–21.17	27.55	26.84–28.26
SRH				
Good	19.09	18.97–19.21	23.23	23.11–23.36
Poor	20.78	20.54–21.01	24.58	24.35–24.81
**Multimorbid**				
No	19.72	19.6–19.84	24.04	23.91–24.16
Yes	18.45	18.24–18.67	22.19	21.97–22.41
**ADL functioning**				
High	19.07	18.96–19.19	23.13	23.01–23.26
Low	21.25	20.98–21.52	25.03	24.79–25.27
**IADL functioning**				
High	18.42	18.29–18.54	22.19	22.03–22.35
Low	21.52	21.33–21.71	24.89	24.74–25.04
**MPCE quintile**				
Poorest	20.81	20.56–21.06	25.21	24.98–25.45
Poorer	20.18	19.95–20.42	24.22	23.98–24.45
Middle	19.39	19.16–19.62	23.68	23.43–23.92
Richer	19.06	18.83–19.29	22.84	22.59–23.09
Richest	17.77	17.54–17.99	21.71	21.44–21.98
**Religion**				
Hindu	19.29	19.16–19.41	23.52	23.39–23.65
Muslim	19.25	18.96–19.53	24.61	24.31–24.92
Christian	20.42	20.07–20.77	22.87	22.48–23.25
Others	20.01	19.53–20.5	22.85	22.35–23.35
**Caste**				
SC/ST	21.13	20.94–21.32	25.23	25.04–25.42
OBC	19.22	19.05–19.39	23.37	23.19–23.55
Others	17.94	17.75–18.12	22.07	21.87–22.28
**Residential status**				
Urban	17.07	16.91–17.24	20.87	20.69–21.06
Rural	20.65	20.52–20.78	25.1	24.98–25.23
**Region**				
North	19.09	18.85–19.32	23.74	23.49–23.98
Central	19.84	19.56–20.11	24.95	24.68–25.22
East	19.66	19.41–19.9	24.35	24.1–24.61
Northeast	20.02	19.69–20.34	23.57	23.24–23.91
West	18.94	18.71–19.17	22.12	21.89–22.36
South	19.48	19.18–19.77	23.45	23.14–23.76
Total	19.43	19.32–19.53	23.55	23.44–23.66

*CI* Confidence interval, *ADL* Activities of daily living, *IADL* Instrumental activities of daily living, *MPCE* Monthly per capita consumption expenditure, *SC/ST* Scheduled caste/Scheduled tribe, *OBC* Other backward classes.

### Moderated multivariable regression estimates of cognitive impairment among older adults

Table 3 provides the linear regression estimates of cognitive impairment in the study sample. Older adults who had depressive symptoms were significantly more likely to have cognitive impairment [uCoef: 0.39, CI: 0.37 to 0.41] in comparison to older adults with no depressive symptoms. The association remained same after adjusting for all the socioeconomic and health-related variables in the study [aCoef: 0.18, CI: 0.16 to 0.20]. Older individuals who were religious were significantly less likely to have cognitive impairment [uCoef: − 1.51, CI: − 1.72 to − 1.30] than their non-religious counterparts. The results were little attenuated but the association remained same after adjusting for all the covariates [aCoef: − 0.43, CI: − 0.61 to − 0.25]. Similarly, compared to older adults who did not participate in religious activities, those who participated in all religious activities after adjusting for other variables had lower likelihood [aCoef: − 0.52, CI: − 0.69 to − 0.34] of cognitive impairment in the current study.

**Table 3:** Moderated multivariable linear regression estimates of cognitive impairment with individual, health and household characteristics among older adults.

Variables	uCoef (95% CI)	Beta	aCoef (95% CI)	Beta	aCoef (95% CI)	Beta	aCoef (95% CI)	Beta
					Model 1		Model 2	
**Depressive symptoms**								
No	Ref		Ref					
Yes	0.39*** (0.37 to 0.41)	0.21	0.18*** (0.16 to 0.20)	0.10				
**Religiosity**								
No	Ref		Ref				Ref	
Yes	− 1.51*** (− 1.72 to − 1.30)	− 0.09	− 0.43*** (− 0.61 to − 0.25)	− 0.02			− 0.43*** (− 0.61 to − 0.25)	− 0.02
**Religious participation**								
No	Ref				Ref			
Yes	− 1.70*** (− 1.91 to − 1.49)	− 0.10	− 0.52*** (− 0.69 to − 0.34)	− 0.03	− 0.51*** (− 0.68 to − 0.33)	− 0.03		
**Depressive symptoms # Religiosity**								
No depressive symptom # Not religious					Ref			
Depressive symptoms # Not religious					0.20*** (0.18 to 0.22)	0.25		
Depressive symptoms # Religious					0.18*** (0.16 to 0.20)	0.23		
**Depressive symptoms # Religious participation**								
No depressive symptom # No religious participation							Ref	
Depressive symptoms # No religious participation							0.19*** (0.17 to 0.20)	0.24
Depressive symptoms # Religious participation							0.16*** (0.14 to 0.18)	0.20
**Age (in years)**								
60–69			Ref		Ref		Ref	
70–79			1.10*** (0.94 to 1.26)	0.07	1.10*** (0.95 to 1.26)	0.07	1.10*** (0.95 to 1.26)	0.07
80 +			2.60*** (2.34 to 2.86)	0.11	2.60*** (2.34 to 2.86)	0.11	2.60*** (2.34 to 2.86)	0.11
*Sex*								
Male			Ref		Ref		Ref	
Female			1.29*** (1.11 to 1.47)	0.09	1.29*** (1.11 to 1.47)	0.09	1.29*** (1.11 to 1.47)	0.09
**Educational status**								
No education			Ref		Ref		Ref	
Primary			− 3.73*** (− 3.92 to − 3.54)	− 0.21	− 3.73*** (− 3.92 to − 3.54)	− 0.21	− 3.73*** (− 3.92 to − 3.54)	− 0.21
Secondary			− 6.43*** (− 6.63 to − 6.23)	− 0.39	− 6.43*** (− 6.63 to − 6.23)	− 0.39	− 6.43*** (− 6.63 to − 6.23)	− 0.39
Higher			− 8.06*** (− 8.35 to − 7.77)	− 0.35	− 8.06*** (− 8.35 to − 7.77)	− 0.35	− 8.06*** (− 8.35 to − 7.77)	− 0.35
**Marital status**								
Currently in marital union			Ref		Ref		Ref	
Not in marital union			0.63*** (0.46 to 0.80)	0.04	0.63*** (0.46 to 0.80)	0.04	0.63*** (0.46 to 0.80)	0.04
**Living arrangement**								
Alone			Ref		Ref		Ref	
With spouse			0.43* (0.07 to 0.78)	0.02	0.43* (0.07 to 0.79)	0.03	0.43* (0.07 to 0.78)	0.02
Others			0.17 (− 0.14 to 0.49)	0.01	0.18 (− 0.14 to 0.49)	0.01	0.17 (− 0.14 to 0.49)	0.01
**Work status**								
Never worked			Ref		Ref		Ref	
Currently not working			− 0.34*** (− 0.53 to − 0.15)	− 0.02	− 0.34*** (− 0.53 to − 0.15)	− 0.02	− 0.34*** (− 0.53 to − 0.15)	− 0.02
Currently working			− 0.61*** (− 0.82 to − 0.40)	− 0.04	− 0.61*** (− 0.82 to − 0.40)	− 0.04	− 0.61*** (− 0.82 to − 0.40)	− 0.04
Retired			− 0.93*** (− 1.22 to − 0.63)	− 0.04	− 0.93*** (− 1.22 to − 0.63)	− 0.04	− 0.93*** (− 1.22 to − 0.64)	− 0.04
**Physical activity**								
No			Ref		Ref		Ref	
Moderate			− 0.57*** (− 0.83 to − 0.31)	− 0.02	− 0.57*** (− 0.83 to − 0.31)	− 0.02	− 0.57*** (− 0.83 to − 0.31)	− 0.02
Vigorous			− 0.67*** (− 0.87 to − 0.47)	− 0.04	− 0.67*** (− 0.87 to − 0.47)	− 0.04	− 0.67*** (− 0.87 to − 0.48)	− 0.04
Both			− 1.44*** (− 1.87 to − 1.01)	− 0.03	− 1.44*** (− 1.87 to − 1.01)	− 0.03	− 1.44*** (− 1.87 to − 1.02)	− 0.03
**Current tobacco use (smoked/smokeless)**								
No			Ref		Ref		Ref	
Yes			0.05 (− 0.12 to 0.22)	0.00	0.05 (− 0.12 to 0.22)	0.00	0.05 (− 0.12 to 0.22)	0.00
**Heavy episodic drinking**								
No			Ref		Ref		Ref	
Yes			0.89*** (0.57 to 1.21)	0.03	0.89*** (0.57 to 1.21)	0.03	0.89*** (0.57 to 1.21)	0.03
SRH								
Good			Ref		Ref		Ref	
Poor			0.49*** (0.32 to 0.67)	0.03	0.49*** (0.32 to 0.66)	0.03	0.49*** (0.32 to 0.66)	0.03
**Multimorbid**								
No			Ref		Ref		Ref	
Yes			− 0.31*** (− 0.47 to − 0.14)	− 0.02	− 0.31*** (− 0.47 to − 0.14)	− 0.02	− 0.30*** (− 0.47 to − 0.14)	− 0.02
**ADL functioning**								
High			Ref		Ref		Ref	
Low			0.59*** (0.40 to 0.78)	0.03	0.59*** (0.40 to 0.78)	0.03	0.59*** (0.40 to 0.78)	0.03
**IADL functioning**								
High			Ref		Ref		Ref	
Low			0.75*** (0.60 to 0.91)	0.05	0.75*** (0.60 to 0.91)	0.05	0.75*** (0.60 to 0.91)	0.05
**MPCE quintile**								
Poorest			Ref		Ref		Ref	
Poorer			− 0.21 (− 0.42 to 0.00)	− 0.01	− 0.21 (− 0.42 to 0.00)	− 0.01	− 0.21 (− 0.42 to 0.00)	− 0.01
Middle			− 0.36*** (− 0.57 to − 0.15)	− 0.02	− 0.36*** (− 0.57 to − 0.15)	− 0.02	− 0.36*** (− 0.57 to − 0.15)	− 0.02
Richer			− 0.70*** (− 0.92 to − 0.48)	− 0.04	− 0.70*** (− 0.92 to − 0.48)	− 0.04	− 0.70*** (− 0.92 to − 0.48)	− 0.04
Richest			− 1.03*** (− 1.26 to − 0.80)	− 0.06	− 1.03*** (− 1.26 to − 0.80)	− 0.06	− 1.03*** (− 1.26 to − 0.80)	− 0.06
**Religion**								
Hindu			Ref		Ref		Ref	
Muslim			0.31** (0.09 to 0.53)	0.01	0.31** (0.09 to 0.53)	0.01	0.31** (0.09 to 0.52)	0.01
Christian			0.37** (0.10 to 0.65)	0.02	0.37** (0.09 to 0.65)	0.02	0.37** (0.09 to 0.64)	0.02
Others			− 0.25 (− 0.57 to 0.06)	− 0.01	− 0.25 (− 0.57 to 0.06)	− 0.01	− 0.26 (− 0.57 to 0.06)	− 0.01
**Caste**								
SC/ST			Ref		Ref		Ref	
OBC			− 0.84*** (− 1.02 to − 0.67)	− 0.06	− 0.84*** (− 1.02 to − 0.67)	− 0.06	− 0.84*** (− 1.02 to − 0.67)	− 0.06
Others			− 0.75*** (− 0.95 to − 0.56)	− 0.05	− 0.75*** (− 0.94 to − 0.56)	− 0.05	− 0.75*** (− 0.95 to − 0.56)	− 0.05
**Residential status**								
Urban			Ref		Ref		Ref	
Rural			1.51*** (1.35 to 1.67)	0.11	1.51*** (1.35 to 1.67)	0.11	1.51*** (1.35 to 1.67)	0.11
**Region**								
North			Ref		Ref		Ref	
Central			− 0.07 (− 0.32 to 0.18)	− 0.00	− 0.07 (− 0.32 to 0.19)	− 0.00	− 0.07 (− 0.32 to 0.18)	− 0.00
East			− 0.08 (− 0.32 to 0.15)	− 0.00	− 0.09 (− 0.32 to 0.15)	− 0.00	− 0.09 (− 0.32 to 0.15)	− 0.01
Northeast			0.28* (0.00 to 0.56)	0.01	0.28* (0.00 to 0.56)	0.01	0.28* (0.00 to 0.55)	0.01
West			− 0.69*** (− 0.92 to − 0.47)	− 0.04	− 0.69*** (− 0.92 to − 0.46)	− 0.04	− 0.69*** (− 0.92 to − 0.46)	− 0.04
South			1.36*** (1.10 to 1.61)	0.07	1.35*** (1.10 to 1.61)	0.07	1.35*** (1.10 to 1.61)	0.07
**Constant**			19.35*** (18.69 to 20.01)		18.99*** (18.35 to 19.63)		19.26*** (18.60 to 19.92)	
*R-squared*			0.47		0.47		0.47	

*if *p* < 0.05, **if *p* < 0.01, ***if *p* < 0.001; uCoef: Unadjusted regression coefficients; aCoef: Adjusted regression coefficients; Beta: Standardized beta coefficients.

Model 1 and 2 are interaction models, adjusted for all the covariates.

Older adults who had depressive symptoms and were not religious were significantly more likely to have cognitive impairment [aCoef: 0.20, CI 0.18–0.22] than those with no depressive symptom and not religious. However, the strength of the association decreased in case of those with depressive symptoms and were religious [aCoef: 0.18, CI 0.16–0.20]. Similarly, older adults with depressive symptoms and did not participate in religious activities had higher likelihood [aCoef: 0.19, CI 0.17–0.20] of cognitive impairment compared to those with no depressive symptoms and no religious participation. However, the strength of the association decreased in case of older adults with depressive symptoms and participated in religious activities [aCoef: 0.16, CI 0.14–0.18].

### Interaction of religion, religiosity, religious participation and depressive symptoms on cognitive impairment among older adults

**Figure 4:**
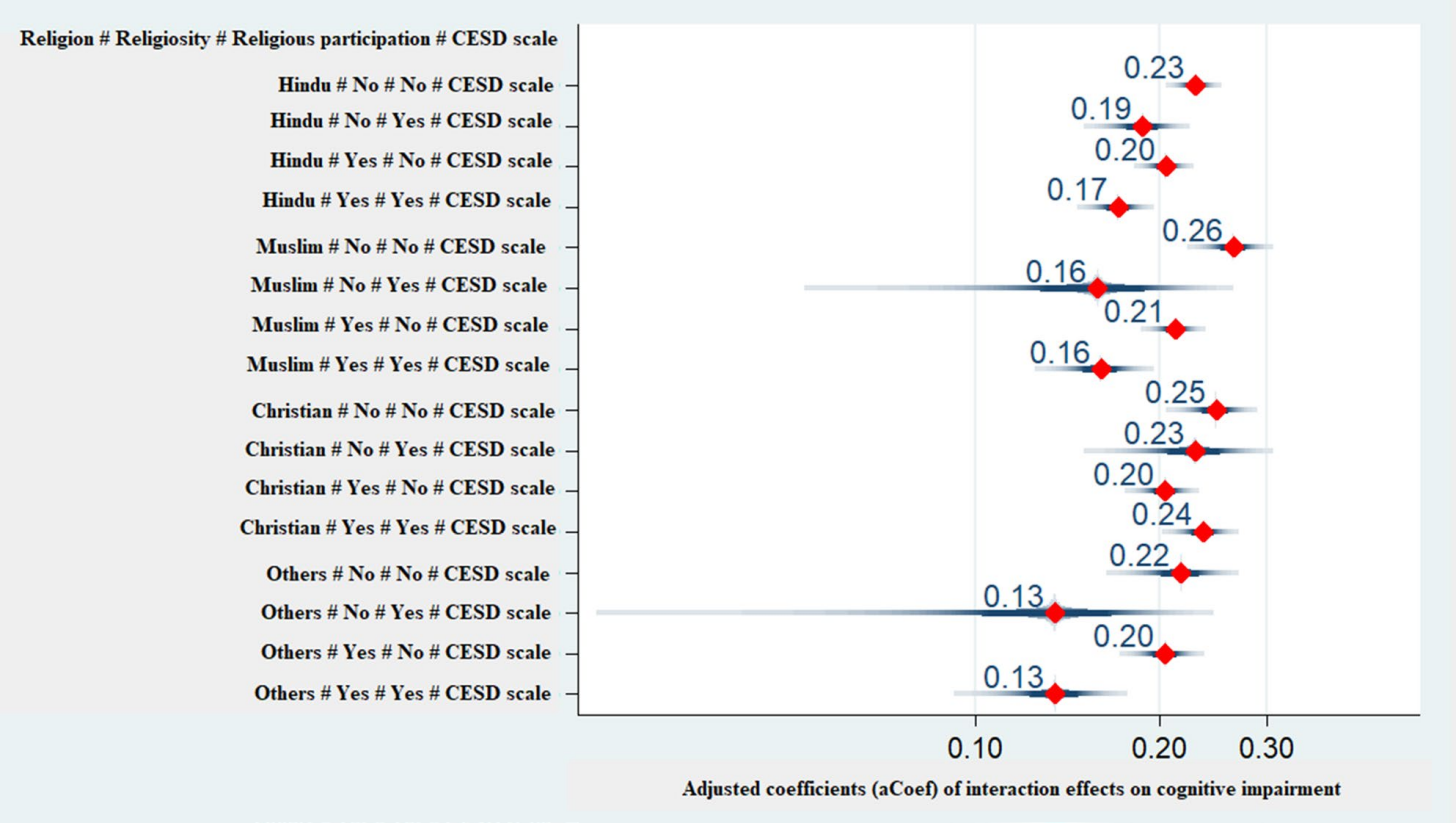
Interaction of religion, religiosity, religious participation and depressive symptoms on cognitive impairment among older adults. *Notes* #: Interaction term; Coefficients are adjusted for the covariates in Table 3; “Others” religious category includes Sikh, Buddhist, Jain, etc. (65 cases of those with no religious affiliation were dropped); CESD refers to the composite score of CES-D scale ranging from 0 to 30; Cognitive impairment scale ranges from 0 to 43. Reference category is religion# not religious# no religious participation# no depressive symptoms.

Figure 4 presents the coefficient plot of moderated linear regression models of interaction between religion, religiosity, religious participation and depressive symptoms on cognitive impairment among older adults (subjects with no religious affiliation (n = 65) were dropped during this analysis). Older adults who were neither religious nor participated in religious activities and had depressive symptoms were more likely to have cognitive impairment compared to those with depressive symptoms who either were religious or participated in religious activities. This was true across all religions and the likelihood was higher in older adults from Muslim religion [aCoef: 0.26, CI 0.23–0.30], followed by Christian [aCoef: 0.25, CI 0.22–0.28] and Hindu religion [aCoef: 0.23, CI 0.21–0.25].

The sensitivity analysis of cognitive impairment scale after removing the suspected dementia cases (those falling in the lowest 10th percentile of composite cognitive functioning score by different educational categories with cut-off score of 14 for illiterate, 18 for primary, 23 for secondary and 26 for higher education) yielded similar results and pointed towards the same conclusions (Table S1 of the Supporting Information file).

## Discussion

Older people participate in religious events more actively than younger people for exchanging reciprocal aids, sharing values and gaining emotional support^64^. The beneficial effects of religious attendance of older adults on their cognitive functioning can be from optimism, having a purpose in the remaining life, salutary values, and the self-efficacy provided by spiritual engagement^65^. Religious involvement would provide older individuals who may be experiencing health deficits with self-improving activities and diverse social networks that improve their mental health outcomes^66,67^. The current study using a nationally-representative survey data of older individuals in India explored the moderating role of religiosity and religious participation on the relationship between depressive symptoms and cognitive impairment.

Several studies have reported that depression is a risk factor for cognitive decline^5,68,69^, and older individuals with depressive symptoms are more likely to suffer from cognitive impairment and dementia^6,70,71^. In line with this, the current study found that older adults who were depressed are more likely to be cognitively impaired after adjusting for several confounding variables. Further, religiosity and religious participation in older people reduced the likelihood of having cognitive impairment in the study. These findings are consistent with a community-based study among the oldest old in China^72^, and a 1 year follow-up study of outpatients with probable mild to moderate Alzheimer’s Disease in Italy^73^, which demonstrated that higher levels of religiosity and religious participation are associated with lower risk of cognitive impairment and dementia and slower cognitive decline. Similarly, a study among older Taiwanese adults found that after adjusting for baseline characteristics, those who engaged in multiple social activities had lesser chances of cognitive failures than those who engaged in none^74^. Furthermore, another study found that religious attendance and private prayer are independently associated with better cognitive health among older adults from different racial and ethnic groups^75^. Multiple studies have also shown that stimulating activities including those as part of one’s religion may help build their cognitive reserve capacity that prevents cognitive deficits or delays the manifestation of cognitive difficulties^29,76–78^.

The current findings can be explained by different mechanisms. Firstly, religious participation is possibly a way to socially engage with people who have the same social background which may enhance their social networks that have positive effects on their mental health. Secondly, as evident in a couple of studies, people with poor socioeconomic background and lower levels of education may rely on religious activities to cope with their distress more than other groups^79,80^. Their feeling of being in socioeconomically disadvantaged group would then make them rely more on religious activities and help them to feel less depressed. In this regard, religious participation may increase sensory stimulation through activities such as prayer, scripture reading, singing, sermons and philosophical discussions. Finally, experiences of comfort, love, and spiritual peace may reduce feelings of anxiety and depression, and may elevate mood and promote optimism and self- esteem, in turn, enhance positive psychological outcomes. This is also supported by previous findings among Korean older adults suggesting that religiosity and spirituality have significant effects on depression and quality of life^81^.

Religiosity certainly plays a role in cognitive functions of older adults, as do religious activities like prayer, however, it is active participation in the religious activities that is important that may create a sense of being engaged with the wider community and has more beneficial effects, rather than merely a sense of being religious^35^. Supporting this, the current findings showed comparatively lower likelihood of cognitive impairment among those with depressive symptoms and were religious than those with no depressive symptoms and were not religious. It is also revealed that older adults with depressive symptoms and participated in religious activities had lower likelihood of cognitive impairment in the study in comparison to their counterparts with no depressive symptoms or not involved in religious activities. This is consistent with a previous finding that greater religious attendance was related to less cognitive decline among older women who were depressed after controlling for socio-economic and demographic variables, health status, physical functioning as well as social support^82^. Similarly, previous research has also shown a moderating effect of church attendance on the relationship between depressive symptoms and cognitive function, where higher levels of church attendance appears to have buffered the negative impact of depressive symptoms on cognitive function^83^. A Chinese study using a structural equation modelling (SEM), found that religiosity partly improves cognitive functioning due to its inverse relationship with depression possibly by increasing social networks^84^.

Another important finding of this paper is the religion and well-being relationship in the context of aging in India and within Indian religions. Given that people in India increasingly turn to their affiliated religion as a source of support while facing any difficulties or mental stress, considerably lower proportion of older sample (0.21%) reported no religious affiliation in this study. The differential effects of religious affiliations on cognitive impairment observed in the interaction results suggest a new arena of future research on the value priorities of religious groups and its influence on individuals’ mental health. The observed variations in the interaction effects of religiosity and religious participation on the relationship between depressive symptoms and cognitive impairment may be partially attributed to the theology and social experience of religious groups, particularly in Indian context with large number of religious subgroups. For instance, liberal religious groups may probably manifest a different pattern on its influence on individuals’ mental wellbeing. Studies that directly address this aspect are required.

Although the findings suggest the positive effects of religiosity and religious participation on mental health of older adults, it should not advocate enhancing the religious engagements that results in social conflicts. Thus, the issue of religious participation and religiosity should be interpreted with care in a country like India, mainly because some groups could misguidedly use such findings to promote their ideologies and create social conflicts. Rather, the findings may suggest that the religious involvements by helping to cope with stressors may help older individuals in maintaining better cognitive functioning. Clinicians may also encourage the depressed older patients who receive antidepressant treatment to go along with faith-based treatment that have well established and showed more efficacious^20^. This may help prevent the larger decline of cognitive abilities by reducing depressive symptoms, thus helping to prevent dementia. The findings may also recommend that older adults with mild cognitive impairment may re-engage in religious activities they may have given up as a result of their depressive symptoms. Further studies need to be conducted to explore the possible pathways in the relationship between depressive symptoms and cognitive deficits after controlling for variables such as political affiliations and neighborhood characteristics of older individuals.

The present study has several limitations. First, the cross-sectional design of this study prevents any statements about the causal direction in the relationships found. Religious involvement could either lead to better cognitive functioning or better cognitive functioning could promote increased religious involvement. Hence, a longitudinal study or randomized trial would be necessary to determine causal relationships. Second, although religious participation is based on multiple questions, religiosity in this study was assessed using a single item. Also, as documented, there is greater wellbeing reported among religious people in religious nations and the specific social setting may be the role-player rather than religiosity per se^85^. Further, although education level was adjusted when examining the relationships between religiosity, depressive symptoms and cognitive impairment, due to the low education level of the sample with a proportion of 74.02% of the total sample being illiterate or having merely a primary level of education, cognitive impairments may have been overestimated producing measurement bias in the current study. However, this is partially addressed in the sensitivity analysis that showed similar findings. Furthermore, the potential confounders including the qualitative aspects of physical, social and religious activities were not included in the current statistical models which could influence relationships between key variables in this study.

## Conclusion

The current study contributes to advancing knowledge about the mental health benefits of religiosity and religious participation by focusing on older adults in India who culturally have limited chances to participate in social activities. The findings suggest that older adults with depressive symptoms may participate in religious activities which may reduce their chances of cognitive impairment. This protective effect of religiosity and religious participation on late life cognitive health has important implications for promoting alternative social support mechanisms for older adults in terms of enhancing their mental wellbeing and contributing to active aging.

## Supplementary Information

Supplementary Information.

## Data Availability

The study used secondary data which is available on reasonable request through https://www.iipsindia.ac.in/content/lasi-wave-i.

## Abbreviations

uCoef: Unadjusted regression coefficients
aCoef: Adjusted regression coefficients
CI: Confidence interval
SRH: Self-rated health
ADL: Activities of daily living
IADL: Instrumental activities of daily living.
MPCE: Monthly per capita consumption expenditure
