# Supplementary material for: The role of religiosity and religious participation in the relationship between depressive symptoms and cognitive impairment among older Indian adults
Source: Sci Rep. 2022 Jul 13;12:11915. doi: 10.1038/s41598-022-14744-3 (PMC9279482; doi:10.1038/s41598-022-14744-3)
Supplement: Supplementary file 1 — Supplementary Information. [file 41598_2022_14744_MOESM1_ESM.docx]

| **Table-S1** Sensitivity analysis of moderated multivariable linear regression estimates of cognitive impairment after removing the suspected dementia cases by individual, health and household characteristics among older adults | | | | | | | | |
| --- | --- | --- | --- | --- | --- | --- | --- | --- |
| **Variables** | **uCoef (95% CI)** | **Beta** | **aCoef (95% CI)** | **Beta** | **aCoef (95% CI)** | **Beta** | **aCoef (95% CI)** | **Beta** |
|  |  |  |  |  | **Model 1** |  | **Model 2** |  |
| **Depressive symptoms** |  |  |  |  |  |  |  |  |
| No | Ref. |  | Ref. |  |  |  |  |  |
| Yes | 0.26*** (0.24 - 0.27) | 0.17 | 0.09*** (0.08 - 0.11) | 0.06 |  |  |  |  |
| **Religiousity** |  |  |  |  |  |  |  |  |
| No | Ref. |  | Ref. |  |  |  | Ref. |  |
| Yes | -0.98*** (-1.18 - -0.78) | -0.06 | -0.16 (-0.32 - 0.01) | -0.01 |  |  | -0.16* (-0.33 - -0.00) | -0.01 |
| **Religious participation** |  |  |  |  |  |  |  |  |
| No | Ref. |  |  |  | Ref. |  |  |  |
| Yes | -1.30*** (-1.49 - -1.11) | -0.09 | -0.38*** (-0.54 - -0.22) | -0.02 | -0.38*** (-0.54 - -0.23) | -0.03 |  |  |
| **Depressive symptoms # Religiosity** |  |  |  |  |  |  |  |  |
| No depressive symptom # Not religious |  |  |  |  | Ref. |  |  |  |
| Depressive symptoms # Not religious |  |  |  |  | 0.10*** (0.08 - 0.12) | 0.07 |  |  |
| Depressive symptoms # Religious |  |  |  |  | 0.09*** (0.07 - 0.11) | 0.08 |  |  |
| **Depressive symptoms # Religious participation** |  |  |  |  |  |  |  |  |
| No depressive symptom # No religious participation |  |  |  |  |  |  | Ref. |  |
| Depressive symptoms # No religious participation |  |  |  |  |  |  | 0.10*** (0.08 - 0.11) | 0.08 |
| Depressive symptoms # Religious participation |  |  |  |  |  |  | 0.07*** (0.05 - 0.09) | 0.05 |
| **Age (in years)** |  |  |  |  |  |  |  |  |
| 60-69 |  |  | Ref. |  | Ref. |  | Ref. |  |
| 70-79 |  |  | 0.73*** (0.59 - 0.88) | 0.05 | 0.73*** (0.59 - 0.88) | 0.05 | 0.73*** (0.59 - 0.88) | 0.05 |
| 80+ |  |  | 1.39*** (1.14 - 1.63) | 0.06 | 1.38*** (1.14 - 1.63) | 0.06 | 1.39*** (1.14 - 1.64) | 0.06 |
| **Sex** |  |  |  |  |  |  |  |  |
| Male |  |  | Ref. |  | Ref. |  | Ref. |  |
| Female |  |  | 1.13*** (0.96 - 1.29) | 0.09 | 1.13*** (0.96 - 1.29) | 0.09 | 1.13*** (0.96 - 1.29) | 0.09 |
| **Educational status** |  |  |  |  |  |  |  |  |
| No education |  |  | Ref. |  | Ref. |  | Ref. |  |
| Primary |  |  | -3.94*** (-4.11 - -3.77) | -0.25 | -3.94*** (-4.11 - -3.77) | -0.25 | -3.94*** (-4.11 - -3.77) | -0.25 |
| Secondary |  |  | -7.01*** (-7.19 - -6.83) | -0.47 | -7.01*** (-7.20 - -6.83) | -0.47 | -7.02*** (-7.20 - -6.83) | -0.47 |
| Higher |  |  | -8.69*** (-8.95 - -8.43) | -0.43 | -8.69*** (-8.95 - -8.43) | -0.43 | -8.69*** (-8.95 - -8.43) | -0.43 |
| **Marital status** |  |  |  |  |  |  |  |  |
| Currently in union |  |  | Ref. |  | Ref. |  | Ref. |  |
| Not in union |  |  | 0.36*** (0.20 - 0.51) | 0.03 | 0.36*** (0.21 - 0.51) | 0.03 | 0.36*** (0.20 - 0.51) | 0.03 |
| **Living arrangement** |  |  |  |  |  |  |  |  |
| Alone |  |  | Ref. |  | Ref. |  | Ref. |  |
| With spouse |  |  | 0.27 (-0.06 - 0.59) | 0.02 | 0.27 (-0.06 - 0.60) | 0.02 | 0.27 (-0.05 - 0.60) | 0.02 |
| Others |  |  | 0.00 (-0.29 - 0.29) | 0.00 | 0.00 (-0.29 - 0.29) | 0.00 | 0.00 (-0.28 - 0.29) | 0.00 |
| **Working status** |  |  |  |  |  |  |  |  |
| Never worked |  |  | Ref. |  | Ref. |  | Ref. |  |
| Currently not working |  |  | -0.05 (-0.22 - 0.12) | -0.00 | -0.05 (-0.22 - 0.12) | -0.00 | -0.05 (-0.22 - 0.12) | -0.00 |
| Currently working |  |  | -0.23* (-0.42 - -0.04) | -0.02 | -0.23* (-0.42 - -0.04) | -0.02 | -0.23* (-0.42 - -0.04) | -0.02 |
| Retired |  |  | -0.50*** (-0.77 - -0.24) | -0.02 | -0.50*** (-0.77 - -0.24) | -0.02 | -0.50*** (-0.77 - -0.24) | -0.02 |
| **Physical activity** |  |  |  |  |  |  |  |  |
| No |  |  | Ref. |  | Ref. |  | Ref. |  |
| Moderate |  |  | -0.44*** (-0.68 - -0.21) | -0.02 | -0.44*** (-0.68 - -0.21) | -0.02 | -0.45*** (-0.68 - -0.22) | -0.02 |
| Vigorous |  |  | -0.58*** (-0.76 - -0.41) | -0.04 | -0.58*** (-0.76 - -0.41) | -0.04 | -0.58*** (-0.76 - -0.41) | -0.04 |
| Both |  |  | -1.01*** (-1.38 - -0.64) | -0.03 | -1.01*** (-1.37 - -0.64) | -0.03 | -1.02*** (-1.39 - -0.65) | -0.03 |
| **Current tobacco use** |  |  |  |  |  |  |  |  |
| No |  |  | Ref. |  | Ref. |  | Ref. |  |
| Yes |  |  | 0.01 (-0.14 - 0.17) | 0.00 | 0.01 (-0.14 - 0.17) | 0.00 | 0.01 (-0.14 - 0.17) | 0.00 |
| **Heavy episodic alcohol** |  |  |  |  |  |  |  |  |
| No |  |  | Ref. |  | Ref. |  | Ref. |  |
| Yes |  |  | 0.73*** (0.44 - 1.01) | 0.03 | 0.73*** (0.44 - 1.02) | 0.03 | 0.73*** (0.44 - 1.02) | 0.03 |
| **SRH** |  |  |  |  |  |  |  |  |
| Good |  |  | Ref. |  | Ref. |  | Ref. |  |
| Poor |  |  | 0.46*** (0.30 - 0.62) | 0.03 | 0.46*** (0.31 - 0.62) | 0.03 | 0.46*** (0.31 - 0.62) | 0.03 |
| **Multimorbid** |  |  |  |  |  |  |  |  |
| No |  |  | Ref. |  | Ref. |  | Ref. |  |
| Yes |  |  | -0.21** (-0.36 - -0.06) | -0.02 | -0.21** (-0.36 - -0.06) | -0.02 | -0.21** (-0.36 - -0.06) | -0.02 |
| **ADL** |  |  |  |  |  |  |  |  |
| High |  |  | Ref. |  | Ref. |  | Ref. |  |
| Low |  |  | 0.24** (0.07 - 0.41) | 0.02 | 0.24** (0.07 - 0.41) | 0.02 | 0.24** (0.07 - 0.41) | 0.02 |
| **IADL** |  |  |  |  |  |  |  |  |
| High |  |  | Ref. |  | Ref. |  | Ref. |  |
| Low |  |  | 0.51*** (0.37 - 0.65) | 0.04 | 0.51*** (0.37 - 0.65) | 0.04 | 0.51*** (0.37 - 0.65) | 0.04 |
| **MPCE quintile** |  |  |  |  |  |  |  |  |
| Poorest |  |  | Ref. |  | Ref. |  | Ref. |  |
| Poorer |  |  | -0.11 (-0.30 - 0.08) | -0.01 | -0.11 (-0.30 - 0.08) | -0.01 | -0.11 (-0.30 - 0.08) | -0.01 |
| Middle |  |  | -0.23* (-0.42 - -0.04) | -0.02 | -0.23* (-0.43 - -0.04) | -0.02 | -0.23* (-0.42 - -0.04) | -0.02 |
| Richer |  |  | -0.45*** (-0.65 - -0.25) | -0.03 | -0.45*** (-0.65 - -0.26) | -0.03 | -0.45*** (-0.65 - -0.25) | -0.03 |
| Richest |  |  | -0.84*** (-1.05 - -0.64) | -0.06 | -0.85*** (-1.05 - -0.64) | -0.06 | -0.85*** (-1.06 - -0.64) | -0.06 |
| **Religion** |  |  |  |  |  |  |  |  |
| Hindu |  |  | Ref. |  | Ref. |  | Ref. |  |
| Muslim |  |  | 0.14 (-0.06 - 0.34) | 0.01 | 0.13 (-0.06 - 0.33) | 0.01 | 0.14 (-0.06 - 0.34) | 0.01 |
| Christian |  |  | 0.26* (0.00 - 0.51) | 0.01 | 0.25 (-0.00 - 0.50) | 0.01 | 0.24 (-0.01 - 0.49) | 0.01 |
| Others |  |  | -0.09 (-0.37 - 0.20) | -0.00 | -0.09 (-0.37 - 0.20) | -0.00 | -0.09 (-0.38 - 0.19) | -0.00 |
| **Caste** |  |  |  |  |  |  |  |  |
| SC/ST |  |  | Ref. |  | Ref. |  | Ref. |  |
| OBC |  |  | -0.52*** (-0.68 - -0.36) | -0.04 | -0.52*** (-0.69 - -0.36) | -0.04 | -0.52*** (-0.69 - -0.36) | -0.04 |
| Others |  |  | -0.40*** (-0.58 - -0.23) | -0.03 | -0.40*** (-0.58 - -0.23) | -0.03 | -0.40*** (-0.58 - -0.23) | -0.03 |
| **Place of residence** |  |  |  |  |  |  |  |  |
| Urban |  |  | Ref. |  | Ref. |  | Ref. |  |
| Rural |  |  | 1.23*** (1.09 - 1.38) | 0.10 | 1.23*** (1.09 - 1.38) | 0.10 | 1.23*** (1.09 - 1.37) | 0.10 |
| **Region** |  |  |  |  |  |  |  |  |
| North |  |  | Ref. |  | Ref. |  | Ref. |  |
| Central |  |  | -0.23 (-0.46 - 0.00) | -0.01 | -0.23 (-0.47 - 0.00) | -0.01 | -0.23 (-0.46 - 0.00) | -0.01 |
| East |  |  | -0.23* (-0.45 - -0.01) | -0.02 | -0.23* (-0.45 - -0.01) | -0.02 | -0.22* (-0.44 - -0.01) | -0.01 |
| Northeast |  |  | -0.32* (-0.59 - -0.04) | -0.02 | -0.31* (-0.59 - -0.04) | -0.02 | -0.31* (-0.58 - -0.04) | -0.02 |
| West |  |  | -0.88*** (-1.10 - -0.67) | -0.06 | -0.87*** (-1.09 - -0.66) | -0.06 | -0.87*** (-1.09 - -0.66) | -0.06 |
| South |  |  | 0.97*** (0.74 - 1.21) | 0.05 | 0.97*** (0.74 - 1.21) | 0.05 | 0.97*** (0.73 - 1.21) | 0.05 |
| **Constant** |  |  | 21.66*** (21.17 - 22.16) |  | 21.53*** (21.05 - 22.01) |  | 21.60*** (21.10 - 22.09) |  |
| **R-squared** |  |  | 0.52 |  | 0.52 |  | 0.52 |  |
| **if p<0.05, **if p<0.01, ***if p<0.001; uCoef: Unadjusted regression coefficients; aCoef: Adjusted regression coefficients; Beta: Standardized beta coefficients* | | | | | | | | |
| *Model 1 and 2 are interaction models, adjusted for all the covariates* | | | | | | | | |
